# What is the financial incentive to immigrate? An analysis of salary disparities between health workers working in the Caribbean and popular destination countries

**DOI:** 10.1186/s12913-019-3896-5

**Published:** 2019-02-08

**Authors:** Gavin George, Bruce Rhodes, Christine Laptiste

**Affiliations:** 1 Health Economics and HIV and AIDS Division, University of KwaZulu-Natal, Durban, South Africa; 2 School of Accounting, Economics and Finance, University of KwaZulu-Natal, Durban, South Africa; 3 HEU, Centre for Health Economics, The University of the West Indies, St. Augustine, Trinidad, Jamaica

**Keywords:** Human resources for health (HRH), Salaries, Caribbean, Migration, Purchasing power parity

## Abstract

**Background:**

The continuous migration of Human Resources for Health (HRH) compromises the quality of health services in the developing supplying countries. The ability to increase earnings potentially serves as a strong motivator for HRH to migrate abroad. This study adds to limited available literature on HRH salaries within the Caribbean region and establishes the wage gap between selected Caribbean and popular destination countries.

**Methods:**

Salaries are reported for registered nurses, medical doctors and specialists. Within these cadres, experience is incorporated at three different levels. Earnings are compared using purchasing power parity (PPP) exchange rates allowing for cost of living adjusted salary differentials, awarded to different levels of work experience for the chosen health cadres in the selected Caribbean countries (Jamaica, Dominica, St Lucia and Grenada) and the three destination countries (United States, United Kingdom and Canada).

**Results:**

Registered nurses in the destination countries, across all experience levels, have greater spending power compared to their Caribbean counterparts. Recently qualified registered nurses earn substantially more in the UK (86.4%), US (214.2%) and Canada (182.5% more). The highest PPP salary ($) gap amongst more experienced nurses (5-10 years) is found within the US, with a gap of 163.9%. PPP salary gaps amongst medical doctors were pronounced, with experienced cadres (10–20 years of experience) in the US earning 316.3% more than their Caribbean counterparts, whilst UK doctors (183.5%) and Canadian doctors (251.3%) also earning significantly more. Large salary differentials remained for medical specialists and consultants. US specialist salaries were 540.4% higher than their Caribbean based counterparts, whilst UK and Canadian specialists earned 95.2 and 181.6% more respectively.

**Conclusion:**

The PPP adjusted HRH salaries in the three destination countries are superior to those of comparable HRH working in the Caribbean countries selected. The extent of the salary gaps vary according to country and the health cadre under examination, but remain considerable even for newly qualified HRH. The financial incentive to migrate for HRH trained and working in the Caribbean region remains strong, with governments having to consider earning potential abroad when formulating policies and strategies aimed at retaining health professionals.

## Background

Developed countries, experiencing an increased demand for health services and an inadequate supply of Human Resources for Health (HRH), have actively recruited health professionals from some of the world’s poorest countries—despite these same countries facing acute health worker shortages of their own [1]. The Caribbean region is contextualized by high migration rates, hindering the delivery of adequate health services [2], and threatening the regions ability to meet objectives set in the Strategy for Universal Access to Health [3, 4].

Aggressive recruiting by destination countries coupled with difficult economic circumstances in source countries create the ideal circumstances for the outflow of labour. For example, the decade of the 1990s and into the 2000s, a time of economic turbulence for Jamaica, saw experienced nurses recruited to the United Kingdom and the United States [5]. Research suggests that over 50,000 nurses have migrated from the Caribbean community, whilst 50% of all physicians trained in Jamaica since 1991 have also migrated [6, 7]. The most popular destination countries for Caribbean HRH include the predominantly English-speaking United States (US), United Kingdom (UK) and Canada, with these countries suffering their own HRH shortages and relying on foreign trained health professionals to augment their health workforce [8]. Whilst the far larger portion of the migrants from the Caribbean are unskilled, there remains inadequate data on migration flows in order to determine the true effect an uncontrolled drain of medical staff may have on the health profile of local populations [9]. Literature has, however, pointed to the dual impact of health worker migration, highlighting not only the erosion of critical skills, but also the lost financial investment in training and educating HRH [3]. As a result of the high cost of training health care workers in developing countries, migration has been perceived as a perverse subsidy by the poorer source countries to the richer destination countries [4].

If skilled health personnel continue migrating, health systems within source countries and the underserved areas in those countries in particular, are likely to be compromised [10]. Policies and strategies are therefore required to arrest the effect of the erosion of HRH to ensure that the domestic health system is able to function optimally. However, in order to adequately develop effective policies which seek to address the migration of Caribbean HRH, there has to be an understanding of the dynamics of health worker employment opportunities, together with the political will to explore strategies aimed at the recruitment and retention of appropriate human resources within the health care system [11]. This policy alignment is essential in offsetting the impact of market dynamics and the resultant mobility of HRH from the Caribbean region [11].

The failure to adequately retain and replenish HRH, either through the continued production of trained health personnel, and retention of HRH, will result in the deterioration of the health service. The prevalent poor working conditions may dangerously perpetuate the out migration of health professionals, with the remaining health professionals motivated to leave the profession or seek opportunities abroad themselves [12]. Whilst poor working conditions and burnout remain a strong motivator for HRH to migrate [7, 13–15], literature highlights a number of additional factors contributing to migration, including the availability of better remuneration abroad [16, 17, 18]; accompanied by improved standards of living and upward social mobility [19], and better opportunities for professional development [13, 16, 19]. Whilst research suggests that the lure of better salaries is the biggest factor contributing to the migration of Caribbean HRH [16, 20], little exists on the salary differentials between HRH in the Caribbean in relation to HRH in the US, UK and Canada. Literature has gone on to suggest that the real motivator of migration is the actual gap between the potential migrants’ current salary level and the level they could potentially attain abroad, positing that the larger the gap, the greater the incentive to migrate [21]. Identifying salary disparities through examining only exchange rates however, limits our understanding of the true financial advantages of migration. The cost of living varies across destination countries thereby requiring an analysis of wages and their purchasing power in order to fully determine the extent of the financial motive to migrate. This study evaluates the strength of foreign salaries by using a PPP based method to equalise the cost of living between selected destination and Caribbean countries. The use of the PPP method has previously been used by Governments to adjust staff salaries whilst literature has advocated for the adoption of the PPP method in studies involving multi-country comparisons of salaries [22].

## Methods

The method for this paper closely follows that of pre-existing work [23–25] and as such the theory of purchasing power parity and their applicability for international salary comparisons is well documented. In sum, PPP exchange rates between two national currencies, are those that equalise the cost of living between those two countries. This provides a more realistic comparison of salaries between countries as a better alternative to using market based spot exchange rates. As such a PPP based salary comparison is more informative for potential migrating labour.

The IMF calculates its PPP estimates based on the basket of goods selected by the International Comparison’s Program (ICP) [26]. The World Bank designed a basket of products for household consumption called the “Global List” and from that the Economic Commission for Latin America and the Caribbean (ECLAC) generated a “Regional List”, according to the characteristics of the region. This was to ensure ‘representativeness’ and ‘comparability’ across participant countries [27]. The latter criteria can involve some technical trade–offs because not all the same products are available in all countries. The task of the ICP is to compare prices; thus requiring goods and services that could be found in all countries, even if they were not fully representative of the consumption in each participant country [27]. Based on this criteria, in the case of the Caribbean, the ICP uses 570 different goods and services, each having an ICP code and a product description [27]. Salary estimates for this paper are from the 2016 or 2017 salary schedules so a comparison on earnings is made using the 2016 purchasing power parity (PPP) exchange rate estimate from the International Monetary Fund [28].

### Data

Salaries are reported in a series of three tables for nurses, doctors and specialists, respectively. For the latter category it was not possible to distinguish between different specialisations [29, 30] for the Caribbean countries. For the migrant country comparisons at the specialist level, an average salary calculated from two of the more highly paid, common and familiar areas of specialisation were chosen: anaesthetist and general surgeon. These specialists all have an early career starting point and can generally be considered consultants as the years of experience increase. It was generally found that the salaries of these two specialists were comparable across the selected destination countries.

Each table presents the basic salary in a national currency unit (NCU), the US dollar equivalent and then the PPP adjusted salary in US dollars. Finally, percentage gaps between country PPP salaries, using the selected Caribbean country figures as a base, is presented as a comparator because these take account of the cost of living between interspatial salaries.

The basic salaries that are reported exclude any weightings based on location and other allowances or benefits. Some countries have more location based salary variation than others. Indeed any lower and upper bound estimates were reduced to a single point average. Whilst it is acknowledged that the ability to earn performance benefits will vary across countries and experience, these would only accrue to specific individuals under specific circumstances and were not considered in this study.

The basic, before tax salary is reported below, including any reported weightings based on location and other allowances or benefits. The dollar market exchange rates and corresponding PPP ratios are then applied to these figures. Tax rates vary across countries but preliminary post tax comparisons with and without PPP application did not change the rankings in the results.

#### Caribbean region

Based on data availability, the selected countries in the Caribbean region for this study included Jamaica, Grenada, Dominica and St Lucia. In terms of HRH qualifications needed to practice as a nurse or doctor and the required steps to be promoted to higher salary levels, the requirements of both are so similar across the island nations that a country by country breakdown within this region was deemed not useful. Rather a more general description of qualifications and experience is sufficient. For instance, a registered nurse in the Caribbean region requires a Bachelor’s of Science/Diploma in Nursing from an accredited institution and must be registered with the relevant nursing council or medical accreditation body [31–34]. Training entails a three year general nursing program, twelve months of midwifery and one year on a nursing assistant program [35].

A medical officer (MO) is generally required to have a Bachelor of Medicine and Bachelor of Surgery (MBBS) or equivalent medical degree recognised by the relevant medical council as well as at least two years of post-internship working in a hospital, preferably including some accident and emergency experience [36–39]. Specialists and consultants often require a postgraduate qualification in their chosen field of expertise and a practicing certificate [40]. This needs to be supported by at least two, sometimes five years of work experience in the specialist field as well as five years of experience in a clinical setting or general practice [18, 19].

Data for St Lucia and Grenada were gathered from Government publications detailing salaries and increases from promotions and career progression [41, 42]. In the case of Jamaica, data were retrieved from the Compensation Unit (responsible for public sector job evaluations and wage policy), operating within the Ministry of Finance and Public Service, which publishes HRH salary scales, including details of career progression and subsequent salary increases [43]. Data on Dominica HRH salaries were considered less reliable, with only ‘estimates’ for the 2016/2017 period found and less clarity on career progressions resulting in salary increases [43]. Nurses generally start on scale point 19 and move to scale 12 after approximately 5 years [44]. It was not clear if they remained on this latter scale significantly beyond 5 years so the most conservative approach (no movement) was adopted. A similar situation emerged for medical doctors. This is illustrated in the results tables for these two HRH categories for Dominica. The final consultant/specialist category did not require such disaggregation.

#### United Kingdom

The UK data for all the HRH categories was gathered using NHS sources [41, 42] which have been used in previous studies analysing HRH salary data [23, 24]. Scales were revised in April 2016 under the ‘Agenda for Change’ and is based on a points system within different bands [41]. In addition, there is also a weighting for HRH working in London [41]. Early career nurses start in band 4, point 15 [45]. Early career, or junior doctors, are paid both a basic salary and a band supplement, which varies on the hours worked [46]. Foreign trained nurses, outside of the European Economic Area (EEA), will need to register with the Nursing and Midwifery Council which will then compare their training with that required by the UK. This is regardless of working within the public or private healthcare sector, with the registration process completed following an examination [47].

In the UK, a junior doctor requires at least Foundation 2 as a career starting point [48]. In some countries a resident medical officer (RMO) is a junior doctor in training, whilst in the UK, the term RMO generally refers to a doctor in a private hospital [49]. Foreign trained doctors outside the EEA will need to register and get a UK license from the General Medical Council (GMC) regardless of whether they work in the public or private health sector. The GMC prescribes its own criteria to determine whether a doctor is fit to practice in the UK [50].

The National Careers Service database was used to determine salaries for the selected UK based specialists [51–53]. Anaesthetists and general surgeons working in the NHS generally require a 5-year degree in medicine, a 2-year foundation program of general training, 6–8 years of specialist training and in the case of surgeons, 2 years of core surgical training and they both receive a similar level of pay [52, 53].

#### United States

Payscale.com, supported by the 2017 Medscape report on Compensation for Physicians, provided the data for all the HRH categories for the United States and accounted for the large salary variation across the country [54–58]. Entry level nurses were found to have the highest variation with a lower and upper bound of US$41,000 – US$70,000 respectively. New York, California, Boston and Seattle can be 30–50% above national average whereas Indianapolis and St Louis are 8 and 14% below the national average [59].

Generally a registered nurse working in the US requires an Associate of Science (ASN) in Nursing or a Bachelor of Science (BSN) in Nursing and a pass in the National Council Licensure Examination (NCLEX) [57, 58]. Licensing and qualification requirements do vary across the country as they do in Canada [60]. Similarly, salaries vary across the country with a national average reported in the data. Doctors in the US are required to have a 4-year undergraduate degree, 4 years of medical school training, 3–7 years of residency training and must have passed the Medical Licensing Examination (USMLE) [61, 62]. Foreign applicants need, inter alia, a license from the state in which they intend to work, pass the first two steps of the USMLE, get into an accredited US or Canadian based residency program then return to pass stage three of the USMLE, all of which can take several years [63]. This also applies to specialists. Anaesthetists and general surgeons require graduate training that leads to a medical degree, followed by 4 years of residency training, although the residency may be longer for surgeons [64, 65].

#### Canada

Payscale.com was used to determine Canadian HRH salaries, again due to the large variation across the country [66]. In particular, nurses operating in remote areas can potentially command considerably higher salaries, and as such a median is reported to remove outlier influence on the mean [67]. Historically, Canadian provinces only required a diploma as a minimum entry requirement into nursing but since 1998 (for the Atlantic provinces at least), nurses require a Bachelor’s degree and the subsequent nursing training can then be completed within 2 to 4 years [68]. Potential applicants do not apply to a central body but rather to a specific facility/hospital [69]. Provinces have their own entry and licensing requirements with variation found at the city level where, for example, a nurse applying to a hospital in Quebec must be proficient in both French and English [69].

Once prospective doctors working in Canada have passed the Medical Council of Canada Evaluating Examinations (MCCEE – parts 1 and 2), the Medical Council of Canada awards a qualification known as the Licentiate of the Medical Council of Canada (LMCC), which allows the graduate to practice medicine in Canada [70]. For specialists such as anaesthetists and surgeons, in addition to the standard medical training, they must pass the Royal College of Physicians and Surgeons of Canada (RCPSC) examinations [71]. Whilst there is considerable regional variation, the Canadian Medical Council has set up the National Assessment Collaboration (NAC) creating a pan-Canadian model that sets common standards, tools and materials for Practice-Ready Assessment (PRA) programs across Canada. These PRA programs are designed to evaluate international medical graduates and foreign trained medical practitioners wanting to practice in Canada. This is to ensure that all PRA programs operate in a consistent and comparable manner across provinces and territories [72] .

A distinction could not be made between private and publically employed consultants/specialists and only general averages were obtained. A large proportion of Canadian specialists, depending on the province, are private practitioners who have their own expenses, revenues, assets and overheads [73, 74]. Petch et al., (2012) found that accounting for overhead charges ‘substantially’ affects income estimates. Estimates reveal that these can be between 12.5% for emergency medicine to 42.5 for ophthalmology (based in Ontario), varying by speciality [75]. Whilst we acknowledge this issue, the results below were not adjusted for any possible overhead charges that consultants/specialists may wish to levy on their clients.

## Results

Tables 1-3 reveal that the different categories of HRH operating abroad have greater spending power than their Caribbean counterparts. Newly or recently qualified registered nurses earn significantly more in the UK (86.4%), US (214.2%) and Canada (182.5%) (Table 1). The salary gap of registered nurses with 5–10 years’ experience is not as wide as their entry level colleagues. The highest (and most enticing) PPP gap can be still found in US with a 163.9% increase for nurses with 5–10 years of work experience. Canada is the next highest at 140.1% and the UK at 92.1% which still promises a great deal more spending power compared with nurses working in the Caribbean countries. For the most experienced nurses, the UK offers the top percentage PPP increase over their Caribbean counterparts at 164.5%. Next highest is the US and Canada at 153.6 and 133.8% respectively.

**Table 1:** Registered nurse salaries of selected countries for three levels of work experience

		Jamaica^5^	Dominica^8^	St Lucia^11^	Grenada	UK	US^20^	Canada^21^
0-3 yrs	NCU^1^	1,120,696	27,032	42,063^11^	27,888^14^	21,692^17^	52,252	59,660
	XCD^2^	23,764	27,032	42,063	27,888	80,043	142,125	122,900
	US ($)^3^	8737	9938	15,464	10,253	26,846	52,252	45,099
	PPP ($)^4^	13,091	14,891	23,171	15,362	30,989	52,252	46,976
	% PPP ($) gap over Caribbean average (16,629)					86.4	214.2	182.5
5–10 yrs	NCU	1,764,916^6^	37,451	47,357^12^	43,884^15^	30,764^18^	60,364	69,084
	XCD	37,424	37,451	47,357	43,884	113,517	164,190	142,313
	US ($)	13,759	13,769	17,411	16,134	38,073	60,364	52,223
	PPP ($)	20,615	20,630	26,087	24,174	43,948	60,364	54,397
	% PPP ($) gap over Caribbean average (22,877)					92.1	163.9	140.1
10–20 yrs	NCU	2,575,035^7^	37,451	53,029^13^	50,724^16^	49,934^19^	68,393	80,085
	XCD	54,602	37,451	53,029	50,724	184,255	186,029	164,975
	US ($)	20,074	13,769	19,469	18,649	61,798	68,393	60,539
	PPP ($)	30,078	20,630	29,212	27,942	71,334	68,393	63,059
	% PPP ($) gap over Caribbean average (26,966)					164.5	153.6	133.8

^1^salary in own national currency unit

^2^Author calculations. Eastern Caribbean Dollars (XCD) using average 2016 market rate [85]

^3^Author calculations. US dollars using average 2016 market rate [85]

^4^Author calculations. International dollar purchasing power parity (PPP) [86]

^5^ [43]

^6^Mid-point of grade 4

^7^End of grade 6

^8^ [43] Scale point 19

^9,10^ Scale point 12

^11^ [87]. Starting salary on grade 9

^12^mid-point of grade 10

^13^ end point of grade 11

^14^ [88]. Starting on grade G

^15^point 5 of grade H

^16^point 5 of grade I

^17^Corresponds to Band 4, point 15 [41, 45]

^18^ Band 6 [41, 45]

^19^Band 7 to 8c [41, 45]. Directors of nursing can earn an additional 50% more [89]

^20^Variation is large for the US as reported above [59]

^21^Canadian nurses start at step 1, mid-career (5–10 years is step 3) with some regional variation especially due to remote region allowances [67]

**Table 2:** Medical doctor salaries of selected countries for three levels of work experience

		Jamaica^5^	Dominica^8^	St Lucia^11^	Grenada^14^	UK^17^	US^20^	Canada
0-3 yrs	NCU^1^	2,403,879^5^	51,606^8^	58,322^11^	46,956	28,425	63,984	68,467^21^
	XCD^2^	50,973	51,606	58,322	46,956	104,888	174,036	141,042
	US ($)^3^	18,740	18,973	21,442	17,263	38,562	63,984	51,854
	PPP ($)^4^	28,079	28,428	32,127	25,866	40,607	63,984	53,911
	% PPP ($) gap over Caribbean average (28,626)					41.9	123.5	88.3
5–10 yrs	NCU	2,793,079^6^	57,504^9^	63,615^12^	61,284^15^	40,925^18^	108,387	115,976^22^
	XCD	59,226	57,504	63,615	61,284	151,013	294,813	238,911
	US ($)	21,774	21,141	23,388	22,531	55,520	108,387	87,835
	PPP ($)	32,625	31,677	35,043	33,759	58,464	108,387	91,320
	% PPP ($) gap over Caribbean average (33,276)					43.1	235.8	174.4
10–20+ yrs	NCU	3,255,348^7^	57,504^10^	68,342^13^	61,284^16^	70,018^19^	146,847	157,129^22^
	XCD	69,028	57,504	68,342	61,284	258,366	399,424	323,686
	US ($)	25,377	21,141	25,126	22,531	94,987	146,847	119,002
	PPP ($)	38,025	31,677	37,647	33,757	100,026	146,847	123,724
	% PPP ($) gap over Caribbean average (35,276)					183.5	316.3	251.3

^1^salary in own national currency unit

^2^Author calculations. Eastern Caribbean Dollars using average 2016 market rate [85]

^3^Author calculations. US dollars using average 2016 market rate [85]

^4^Author calculations. International dollar purchasing power parity (PPP) [86]

^5^ [43] Medical Officer level 2 (MO2)

^6^ MO3

^7^ MO4

^8,9,10^ [44] No available data on salary progression

^11^ [87]. Grade 13

^12^ step 3 or 4 of grade 14

^13^ end point of grade 15

^14^ [88]. Grade J at entry level

^15^grade J after 10 years

^16^grade J after 20 years or more

^17^ Foundation yr. 1 and 2 respectively. In the UK a medical officer is generally classed as a junior doctor. [90]

^18^ Doctors continue to work and move onto a specialty [90]

^19^Additions to basic salaries can be substantial through clinical excellence awards [42]

^20^ [54]

^21^The Canadian doctor starter salary is based upon 1–4 years of experience [91]

^22^Additional years of experience could not be clearly identified so the same increases for the US were used as a reasonable proxy [54] but it is acknowledged that some regional variation would apply to Canada [92]

**Table 3:** Consultant/Specialist salaries of selected countries

Consultant/ Specialist		Jamaica^5^	Dominica^6^	St Lucia^7^	Grenada^8^	UK^9^	US^10^	Canada^11^
Entry level to 20 yrs experience	NCU^1^	4,597,824	66,810	74,817	63,420	57,783	270,734	199,627
	XCD^2^	101,946	66,810	74,871	63,420	213,219	736,398	411,232
	US ($)^3^	37,480	24,563	27,506	23,316	78,389	270,734	151,188
	PPP ($)^4^	56,158	36,803	41,214	34,936	82,547	270,734	119,046
	% PPP ($) gap over Caribbean average (42,278)					95.2	540.4	181.6

^1^salary in own national currency unit

^2^Author calculations. Eastern Caribbean Dollars using average 2016 to date market rate [85]

^3^Author calculations. US dollars using average 2016 to date market rate [85]

^4^Author calculations. International dollar (purchasing power parity, PPP) [86]

^5^ [43] MO6 with no promotion to MO7 after 20 yrs.

^6^ [44] No available data on salary progression

^7^ [87] Grade 17 with no promotion to 20 yrs. experience

^8^ [88] Grade k at entry level to 20 yrs. experience

^9^ [52] [53]

^10^ [55] [56] [93]

^11^ [94, 95]

A similar hierarchy and consistency in PPP gaps can be found with regard to medical doctors (Table 2), with US salaries highest across all levels of experience. The salary gap between the US is 123.5% over newly or recently qualified medical doctors working in the Caribbean. Canada offers the next highest (88.3%) followed by the UK (41.9%). Medical doctors with 5–10 years’ experience earn 43.1% more in the UK, with the US revealing the highest income disparity at 235.8% more than their Caribbean counterparts. Canadian doctors earn 174.4% more than doctors in the Caribbean with similar experience. Medical doctors with the highest level of experience can potentially realize the highest PPP adjusted increases. Compared to their Caribbean counterparts, US doctors earn 316.3%, Canadian doctors 251.3% and UK doctors 183.5% more than the PPP adjusted Caribbean salaries at this level.

Salaries of consultants and specialists of selected countries from entry level up to 20 years of experience are shown in Table 3. For the specialist/consultant category the salary trends continue. The US continues to offer the highest salaries at some 540.4% more than their Caribbean based counterparts. Canada is next highest at 181.6%, with the UK a more ‘modest’ offering of 95.2% more than the specialists counterparts working in the Caribbean countries.

## Discussion

This study evaluated HRH salaries of selected health cadres across selected Caribbean countries and compared them with the salaries on offer in three popular destination countries. Analyses of salaries using the PPP method affirms the theory that salaries in destination countries are superior and would potentially act as a ‘pull’ factor for Caribbean based HRH [76]. HRH across the health cadres in the US, UK and Canada enjoy considerably greater spending power than their HRH counterparts in the Caribbean, with the extent of the PPP adjusted salary gap varying between different destination countries and across HRH cadres. Whilst it is acknowledged that these HRH salaries are broad estimates based on average salaries across three health cadres and are subject to considerable variation, nonetheless the results are indicative and remain useful for comparative purposes.

The findings of this research provide the first estimates of actual salary differentials between selected Caribbean HRH and their counterparts in the US, UK and Canada. Previous research has noted that salary gaps exist between source and destination countries [16, 20] without providing actual figures. More recent literature has affirmed the notion of out-migrating workers earning more than their peers who remained in the home country, whilst also enjoying better working and living conditions [77]. In fact, this same study suggested that highly qualified health personnel from abroad, including medical doctors and specialists, could in fact enjoy a small wage premium over their indigenous colleagues [77].

It is however understood that the decision to migrate is not solely based upon salary differentials, however large. We know that a number of factors drive migration whilst there remain other considerations as well, including the challenge of finding work in another country [20]. All destination countries will require formal proof of qualifications and may require some prescreening in the form of examinations and testimonies. This process can be prohibitively costly in terms of time and out-of-pocket expenses.

### Policy recommendations for the Caribbean

Thomas-Hope (2002) has warned to refrain from attempting to ‘manage’ migration by limiting peoples’ ability to move [7], especially in the Caribbean, where there remains a positive association with outward movement and freedom and opportunity. The WHO Code of Practice on the International Recruitment of Health Personnel sets out ethical principles applicable to the international recruitment of health personnel in a manner that strengthens the health systems of developing countries, and mentions small island states specifically [78]. Adherence to this Code, together with bilateral agreements, could potentially ensure the controlled and responsible migration of skills between countries. Examples of bilateral agreements include the UK-South Africa Memorandum of Understanding (MOU) and the Pacific Code and the Caribbean Community (CARICOM) agreement. However, a World Bank report on nurse labour markets in the Caribbean community noted that bilateral initiatives between source and destination countries have, to date, had limited impact due to the different interests and agencies involved [57]. Despite the potential limitations of these arrangements, receiving countries and sending countries need to consider a development policy that places greater emphasis on temporary movement, incentives to return home and on resolving the institutional failures that are resulting in health professionals leaving the Caribbean in pursuit of better economic opportunities [13]. Literature emphasises that whatever policy is introduced, it should be buttressed by accurate migration data that can be used for appropriate decision-making. Murphy et al. (2016) bemoan the fact that the minimal formal tracking of health worker migration from Jamaica specifically, results in the scientific analysis of its consequences of migration difficult [11]. This conclusion can comfortably be applied to other countries in the Caribbean region.

The World Bank has advocated for a number of national policies and strategies that seek to limit the consequences of out migration, including: increasing completion rates of medical students; increasing nurse training capacity; mobilizing inactive HRH; and improving the allocation and efficiency of existing operational HRH. Of concern, the report identified that only 55% of medical students completed their studies within the Caribbean region, rendering the number of drop outs a tremendous loss of potential HRH. What is more, existing infrastructure constraints for nurse training can be reduced if additional finances and more clinical opportunities are provided [46].

Literature has suggested that source countries revisit their wage and incentive structure in light of international disparities [79]. Whilst this study provides valuable salary data which could be used to benchmark HRH salaries, policies on working conditions, wage rates and incentives have had limited effect due to global uneven development, problems of national economic development or intensified recruitment – with even the rare integrated policies proving largely ineffectual [80].

The results of this study should be interpreted against its limitations. This study did not review private health sector salaries which are potentially higher. It was felt that the more useful comparison should be made between the public health sectors in the selected countries, especially Canada where HRH requirements vary across provinces and private specialists may incur significant overhead costs [74]. It should be acknowledged that the private health sector in the Caribbean is playing a significant role in growing the medical tourism industry within the island states and is thought to be playing a positive role in retaining domestically trained HRH and even attracting health personnel from abroad due to the improved salaries on offer [81]. Of course the private sector may also provide an additional employment choice for domestic HRH thereby further compromising the ability of the public health care system to recruit and retain health personnel. Comparing private and public sector based remuneration would be a worthy consideration for future research. Furthermore, this study did not factor in the demand and new technologies affecting the HRH cadres analyzed in this study, and recognizes that the ability of Caribbean trained HRH to find comparable employment abroad will depend on these factors. It is recognized that domestically trained medical students are often drawn into specific specialties due to salary and workload benefits, which in the US has included radiology, ophthalmology, anesthesiology and dermatology (ROAD) [82], resulting in fewer available vacancies for foreign trained specialists to fill. In addition, employment prospects are affected by technological advances, with the increased use of artificial intelligence impacting both the demand and salary of radiologists in the US for example [83].

Lastly, selecting countries which have considerable spatial variation of wages within a particular profession will always introduce some unreliability into the data. Accurately determining levels of experience and the consequence for wage increases can only be made on a general basis and individual cases, in different countries, may depart considerably from general expectations due to other factors that affect their earning potential. All of those effects cannot be captured here. The strength of this study is that wage comparisons were made using PPP ratios provided by the IMF which were calculated from data provided by the ICP [84]. Accounting for differences in the cost of living provides a more insightful investigation compared to just using market rates of exchange. However, it is recognized that the method itself is limited in that the basket of goods and services used to estimate the PPP is unlikely to be fully representative. This has been acknowledged by the ICP and best estimates are always provided [27]. The consequences of inaccuracy would be to change the PPP ratio but this is not expected to result in vast differences and the results would not change significantly.

## Conclusion

This study contributes to a greater understanding of the extent of the salary disparity between the Caribbean and popular destination countries across HRH cadres. The financial incentive for Caribbean HRH to seek work abroad remains strong when considering differences in the cost of living, even for HRH with little career experience. Governments therefore have to consider the earning potential abroad when formulating policies and strategies aimed at retaining health professionals.

## Abbreviations

ASN: Associate of Science
BSN: Bachelor of Science (Nursing)
CARICOM: Caribbean Community
CMA: Canadian Medical Association
CSME: Caribbean Single Market and Economy
HRH: Human Resources for Health
LMCC: Licentiate of the Medical Council of Canada
LMIC: Lower Middle Income Countries
MBBS: Bachelor of Medicine and Bachelor of Surgery
MCCEE: Medical Council of Canada Evaluating Examinations
MOU: Memorandum of Understanding
NCLEX: National Council Licensure Examination
NCU: National Currency Unit
NHS: National Health Service
PPP: Purchasing Power Parity
RCPSC: Royal College of Physicians and Surgeons of Canada
RMO: Resident Medical Officer
SGU: St. George’s University
TAMCC: T.A. Marryshow Community College
USLME: United States Medical Licensing Examination
XCD: Eastern Caribbean Dollar
